# Gaming among Children and Adolescents during the COVID-19 Lockdown: The Role of Parents in Time Spent on Video Games and Gaming Disorder Symptoms

**DOI:** 10.3390/ijerph18126642

**Published:** 2021-06-21

**Authors:** Maria Anna Donati, Cristiana Alessia Guido, Giuliano De Meo, Alberto Spalice, Francesco Sanson, Carola Beccari, Caterina Primi

**Affiliations:** 1 NEUROFARBA Department, Psychology Section, University of Florence, 50135 Florence, Italy; francesco.sanson@unifi.it (F.S.); carola.beccari@unifi.it (C.B.); caterina.primi@unifi.it (C.P.); 2 Department of Maternal Sciences, Child Neurology Division, Sapienza University of Rome, 00185 Rome, Italy; cristiana.guido@uniroma1.it (C.A.G.); giuliano.demeo@live.it (G.D.M.); alberto.spalice@uniroma1.it (A.S.); 3 Department of Developmental and Social Psychology, Faculty of Medicine and Psychology, Sapienza University of Rome, 00185 Rome, Italy

**Keywords:** children, adolescents, video gaming, gaming disorder, COVID-19, parents, path analysis

## Abstract

It is mainly children and adolescents who are involved in video gaming. The lockdown caused by the COVID-19 pandemic may have further increased their use of video games and, consequently, the risk of gaming disorder (GD) symptoms. However, currently, we do not have exhaustive knowledge of this issue. To fill this gap, the current study aims to analyze video gaming habits in children and adolescents during the lockdown, starting in March 2020 in Italy, the first European country affected by the pandemic. Specifically, we aim to understand how variables related to parents—for instance, knowledge of their offspring’s life, the monitoring of their video gaming habits, and parental use of video games—are related to their offspring’s time spent on video games and GD symptoms. A web-based survey involving parents (*n* = 554, 79% mothers, mean age = 45.39) of 554 children and adolescents (73% males, mean age = 11.11) was utilized. The results showed that they were involved in video games, particularly boys and adolescents, with high rates of GD symptoms. The parents also spent a considerable amount of time playing video games. A path model that explained the mechanisms through which parental variables were related to their offspring’s time spent on video games and GD symptoms, controlling for gender and age, was verified. Overall, the findings indicate the importance of educating parents to behave effectively with respect to video games and monitor their offspring’s video gaming habits.

## 1. Introduction

The popularization of the internet and the invention of smartphones has had a significant impact on the use of various types of electronic devices for video games (VGs). Indeed, the VG industry has shown a very rapid increase over the last decade [1], so much so that in the adult population, video gaming represents a daily activity for a consistent percentage of adults [2], with a prevalence of VG use of about 56% [3] and an average time of use of about 9 hours per week [4].

The prevalence of video gamers among the juvenile population seems to be even higher, as reported by several studies, indicating that around 90% of adolescents use VGs [5], with the average time spent on video gaming estimated to be between 11.3 [6] and 13.2 h a week [7]. Overall, there is a large body of literature attesting to a wide involvement of adolescents in video gaming [8,9,10,11]. Additionally, children are increasingly involved in VGs [6,7,12,13,14,15,16,17], with research showing that around 90% of children are gamers [12,15], some even starting from the early age of 6 [18,19], with children aged 9–11 spending about 12.7 h per week on VGs and children aged 12–14 years playing for 17.2 h per week [6]. Overall, males have been found to play VGs more frequently than females [20,21], and adolescents seem to spend more time on VGs in comparison to children [22]. However, with regard to the measurement of video game habits in youths, it is important to underline that in some cases [21,23], parental perceptions of their offspring’s behavior have been collected, rather than relying on the children’s own self-reported evaluation, without offering psychometric evidence about their adequacy.

These prevalence data could have further increased during the lockdown imposed by the coronavirus (COVID-19) pandemic, officially declared as such on 11 March 2020 by the World Health Organization (WHO). In fact, as COVID-19 is transmitted between humans in close proximity, preventive measures, such as social distancing and quarantines, have been employed to prevent the spread of the disease. These “stay-at-home” measures provoked a spread of indoor activities, especially electronic video game playing [24,25], which is typically a solitary home pastime [26]. Indeed, it has been found that 82% of global consumers played VGs and watched video game content during the height of the lockdown period of the pandemic [25]. Moreover, increased web-based gaming was viewed as complementary to public efforts to promote physical distancing [27]. In particular, the WHO partnered with the gaming industry in March 2020 to launch the campaign #PlayApartTogether to encourage people to stay at home, play VGs, and practice physical distancing (https://www.bigfishgames.com/us/en/play-apart-together.html (accessed on 2 March 2021)). Due to these incitements, after the introduction of lockdown and quarantine measures, European mobile game downloads reached a record high (an increase of 19%) in March 2020 [28]. 

Concerns have been raised about encouraging video gaming [27] as the time-consuming nature of playing VGs has been found to induce people, especially adolescents, to play games for an excessive amount of time [29]. Spending persistent and excessive time on playing VGs is a fundamental risk factor for the development of pathological behavioral symptoms [30,31,32,33], which have recently been discussed as a formal psychological disorder by both the American Psychiatric Association (APA) and the WHO. In 2013, the APA included *Internet Gaming Disorder* (IGD) in the fifth revision of the *Diagnostic and Statistical Manual of Mental Disorders* (DSM-5) [34]. This disorder is characterized by five or more criteria that must be present over a period of at least 12 months. These criteria include symptoms such as concern (or salience), specifically fixed thoughts on gaming activity, abstinence (generally described as irritability, anxiety, or sadness) when video gaming is interrupted or when the person tries to stop playing, and tolerance (the need to spend increasing amounts of time playing to achieve the desired effects). More general in its formulation, *Gaming Disorder* (GD) has been included as a formal diagnostic entity in the 11th edition of the *International Classification of Diseases* (ICD-11) [35] in the section on addictive disorders in the mental health chapter [36]. GD refers to persistent or recurring gaming behavior (be it a digital or video game that can be implemented in offline or online mode). It is a disorder characterized by three inclusion criteria: loss of control in the game; the growing priority that is given to this activity that determines the predominance of the game over other interests of life and daily activities; and the continuation or escalation of the conduct of play despite the occurrence of negative consequences.

Data regarding the prevalence of GD symptoms pre-COVID-19 are different due to the use of assessment tools with different theoretical and empirical backgrounds and cut-off values. However, some nationally representative surveys have been conducted with self-report measures, and almost all have analyzed teenagers. With regard to adolescents, the prevalence rates were as follows: 1.7% in Germany [37]; 4.2% in Norway [38]; 4.6% in Hungary [39]; 1.3% in the Netherlands [40]; 8.5% in the United States [7]; and 9% in Singapore [13]. In addition, a transnational European survey comprising seven countries [9] reported the following prevalence data: 0.6% in Spain; 1% in the Netherlands; 1.3% in Romania; 1.6% in Germany; 1.8% in Iceland; 2% in Poland; and 2.5% in Greece. Concerning children, GD results to affect 9% [13]. Gender differences should be underlined, as males exhibited more GD symptoms than females [41,42], regardless of age.

Given this premise, it is important to further analyze VG use during the COVID-19 lockdown, with particular emphasis on the juvenile population in relation to both adolescents and children. Indeed, school closure and event cancellations limited their social interactions during this pandemic. Consequently, the risk for the development of GD symptoms may have increased because of the enhanced opportunity to play VGs and decreased access to alternative social activities [43,44]. Moreover, in this specific and special context of domestic quarantine, the risk or protective role of parents with respect to their offspring’s video gaming behavior can also be more precisely analyzed, as a large proportion of the adult population either stopped working, reduced their working hours, or worked from home due to the COVID-19 [45]. Some studies have already conducted on this issue. For instance, a longitudinal study conducted in China attested that both adolescents and children increased their video game use during the COVID-19 pandemic, with adolescents experiencing significantly increased GD symptoms [46]. Another longitudinal study in Germany on adolescents from 10 to 17 years old suggested that usage frequencies significantly increased under the lockdown compared to before the COVID-19 pandemic [47]. A retrospective study conducted in Hong Kong on children and adolescents reported that, overall, 83% of the participants played video games during the COVID-19 pandemic, with a prevalence of 20.9% of excessive gamers and 5.3% of pathological gamers [48]. All these data seem to support suggestions that the COVID-19 pandemic will lead children and adolescents to be more engaged with playing video games.

Following these premises, the general goal of this study is to exploit the lockdown period in Italy (the first European country to be affected by the virus in March 2020 and the first to adopt restrictive measures of social distancing) to more deeply understand video gaming behavior in children and adolescents in order to gain information regarding gaming prevention. To the best of our knowledge, only one previous study has been conducted on video gaming in juveniles in Italy during the COVID-19 pandemic, focusing only on children aged from 8 to 10 years old [49]. This study showed that 96% of children played video games; however, it was conducted between September and November 2020, thus during the second wave of the pandemic, when there was only a partial lockdown, without school closures, and the sample size was relatively small. Our aim was not only to describe video gaming behavior in children and adolescents but also to develop and test a model to explain GD symptoms in this specific time frame that takes into account the role of parents. In this regard, although GD must be read as a multidimensional phenomenon that can be explained by different factors, such as game-related, individual, and environmental factors [30], the role of parents in their offspring’s behavior has not yet been adequately analyzed [50]. 

First of all, generally speaking, the parent–child relationship is particularly important as some studies have found that poor quality parent–child relationships are related to excessive video gaming and pathological behavior [51] as there are low levels of openness or communication between the parent and the child [52]. Moreover, through the analysis of longitudinal data [53], the parent–child relationship has been found to be a negative predictor of pathological symptoms of video gaming in offspring. Related more to the use of VGs, a central dimension to be considered is how parents monitor and control the offspring’s video game playing [29], although research on parental mediation of children’s and adolescents’ use of VGs is still scarce [54]. Broadly speaking, we know that greater general parental monitoring of adolescents is associated with less teen engagement in multiplayer online games [42]. More specifically, setting restrictions on the time, place, and content of video gaming to prevent excessive gaming or reduce symptoms seem to be effective strategies to monitor the offspring’s time spent on VGs [55]. However, the regulating effect of these rules in reducing the amount of time spent on playing games seems to be moderated by the degree of parent–child agreement on the rules [56]. Significant relationships with the offspring’s gender and age have been found concerning the above-cited parental variables. Indeed, the parent–child relationship has been found to be negatively correlated with age among juveniles [57]. Parental monitoring of youths’ video gaming habits seems to be higher for females [29], with differences across gender [58], and appears to be stronger for children than for adolescents [29,50,58].

Another variable related to parents that could have a role in affecting youth behavior concerning video gaming is the parental use of VGs. Indeed, although there is still poor knowledge regarding this issue, it has been found that parents’ time spent on computers is positively associated with teenagers’ computer time, and parents’ engagement in seven types of internet activity, among them, the playing of online multiplayer games, also predicted the teenagers’ engagement in those activities [42]. More generally, mothers’ and fathers’ media practices were found to be associated with children’s screen-time [57], indicating that parental closeness with sons, parental monitoring of their children’s behavior, and parental behavior itself have a modeling role for their offspring’s habits, relative to the use of VGs. 

In more detail, the aim of this study was twofold. First, we aimed to investigate video gaming behavior in children and adolescents during the lockdown. In particular, we were interested in analyzing the prevalence of VGs and online VGs, gaming habits, preferred video game genres, the frequency of video gaming (time during the day spent on VGs and hours/week dedicated to video gaming), the devices use to game, and the social partners involved in gaming. We were also interested in assessing the prevalence of GD symptoms. All the variables described above were analyzed separately for children and adolescents in order to obtain better evidence on age-related specificities. We also took gender into account in each group given the significant role of gender in video gaming behavior in both children and adolescents [20,21,22,41,42], also in the specific time of the COVID-19 pandemic [47,48,49].

Second, we developed and tested a model to explain GD symptoms in the juvenile population by taking into account some risk and protective factors related to parents. In particular, we considered parental knowledge of their offspring’s life habits, parental video gaming behavior, and parental monitoring of their offspring’s video gaming habits. Scant past research has investigated these variables separately with respect to pathological gaming. The contribution of our study is to develop and test the adequacy of an integrated model in which these parental variables are considered all together with respect to the offspring’s video gaming and pathological gaming behavior. 

In detail, we hypothesized a serial mediation model to explain the mechanism through which these factors are associated with the offspring’s time spent on gaming and GD symptoms. Consistent with prior research [29,58], we predicted that parental knowledge of their offspring’s life and the parents’ time dedicated to gaming would be related negatively and positively, respectively, with parental monitoring of their offspring’s video gaming habits and that the parents’ time dedicated to gaming would be positively associated with the time spent by young people on VGs [42,57]. We also expected that parental knowledge of their offspring’s life and the parents’ time dedicated to gaming would be negatively correlated [29,59]. Moreover, based on past studies [60], we posited that parental monitoring of their offspring’s video gaming habits would be negatively related to time spent by the offspring on VGs, which, in turn, would have a positive direct effect on GD symptoms [30,31,32,33]. We also hypothesized indirect effects from the parental variables to time spent by the juveniles on VGs. Specifically, we predicted indirect effects from parental knowledge of their offspring’s life and the parents’ time dedicated to gaming on time spent by their offspring on VGs in negative and positive directions. Indirect positive effects on GD symptoms were expected as a result of parental knowledge of their offspring’s life and parental monitoring, while a negative indirect effect was predicted as a result of time spent by parents on VGs. In testing the model, we controlled for possible effects of gender and age, given the association between the young person’s age and parental knowledge of the offspring [57], the significant role of gender with respect to parental monitoring [29,58], time spent on gaming and GD symptoms in children and adolescents [21,42,43], and, also, the relationship between the offspring’s age and parental monitoring [42,59], as well as time spent by juveniles on gaming [25].

## 2. Methods

### 2.1. Participants and Procedure

A web-based survey was developed using an online platform. The survey was addressed to parents of children and adolescents from kindergarten to the last grade of high school. The link was made available online on 15 April 2020, when the entire Italian population was in total lockdown. Data were collected for two months. Snowball sampling via social media within Italy was used for data collection. The survey took approximately 30 min to be completed. In a preliminary section, the purpose of the study was explained, and participants had to provide consent in order to participate. Participants could stop the survey at any time, and they could interact with the principal investigators of the study through email at any time during and after study participation. The survey was anonymous, and confidentiality of information was assured according to the provisions of the General Data Protection Regulation (GDPR 679/2016).

The participants consisted of 554 parents (79% female) with a mean age of 45.52 years (*SD* = 5.93; range: 29–70). The offspring (73% males) had a mean age of 11.11 years (*SD* = 3.37; range: 3–19 years). 

With regard to educational levels attained, 10% of the parents had a middle school diploma, 36% had a high school diploma, 37% had a university degree, and 17% had a postgraduate specialization. Concerning marital status, 80% of parents were married, 7% were single, 12% were separated/divorced, and 1% were widowed. With regard to occupational status, 91% were employed, and 9% were unemployed. We classified participants who worked based on the Italian National Institute of Statistics (ISTAT), which divides the population into nine categories. These categories are as follows: legislators, managers, and entrepreneurs (6%); intellectual, scientific, and highly specialized professions (28%); technical professions (9%); employees (25%); business and service professions (22%); artisans, and farmers (4%); plant and semi-skilled workers of fixed and mobile machinery (1%); occupations (3%); armed forces (2%).

With regards to the offspring, 306 were children (3–11 years), and 248 were adolescents (12–19 years). We considered the age of 12 as a starting point for adolescence [60]. Children (69% males) had a mean age of 8.62 (1.91); the responding parent was generally the mother (82%, mean age = 43.09, *SD* = 5.14), and the average number of family members was 3.62 (*SD* = 0.83). Adolescents (77% males) had a mean age of 14.17 (*SD* = 1.94); the responding parent was generally the mother (75%, mean age= 48.23, *SD* = 5.60), and the average number of family members was 3.78 (*SD* = 0.99). 

### 2.2. Measures

Initially, socio-demographic information (age, gender, educational qualifications, occupation, marital status, number of offspring), working conditions (continuation of activity and working methods), and living conditions during the lockdown (square meters of the house, number of cohabitants, presence of open spaces, number of electronic devices in the house) were requested. 

### 2.3. Offspring’s Video Gaming Behavior

Subsequently, parents were asked to indicate socio-demographic information about their son/daughter (age, gender) and to respond to the *Video Gaming Scale for Parents* (VGS-P), a hetero-evaluative instrument derived from the *Video Gaming Scale for Adolescents* (VGS-A) [61] and the *Video Gaming Scale for Children* (VGS-C) [12]. These are two scales aimed at investigating video gaming habits and GD symptoms, as a one-dimensional concept, in adolescents and children, respectively. The VGS-P was developed to be a single instrument, employable on both children and adolescents and to be administered to parents, in line with previous studies [19,23]. Moreover, it allowed us to collect a hetero-evaluation of juvenile video gaming habits in the pandemic period. The psychometric analysis of the scale can be found in Appendix A.

The VGS-P is divided into two sections; the first is related to video gaming habits, while the second is to investigate the symptoms of pathological video gaming based on DSM-5. Both sections referred to the period of lockdown. More specifically, in the first section, parents were asked to report whether their son/daughter had used VGs during the isolation period [*yes*, *no*]. Based on the response, children and adolescents were classified as *video gamers*/*non-video gamers*. Parents were also asked to indicate their son/daughter’s favorite type of VG, classified into different genres [2]. Then, they had to declare how often (*never*, *a few times*, *many times*) their son/daughter used the listed devices (computer, tablet, fixed console, portable console, smartphone) to play VGs. In the scoring phase, options (*“sometimes”* and *“many times”*) were collapsed in order to obtain an affirmative or negative answer regarding the use of devices and a versatility score indicating the number of different devices used to game. Then, parents were asked to report at what time of day (*just woken up, in the morning, at lunch, after lunch, in the afternoon, before dinner, at dinner, before going to bed*) their son/daughter played VGs; in this way, it was possible to detect the prevalence of video gamers at single moments during the day and to obtain a versatility score indicating the number of different moments per day spent on gaming. Moreover, parents were asked to indicate how many *hours a day* and how many *days a week* their son/daughter played VGs. In order to determine the time spent on VGs, we multiplied the hours per day spent on VGs [62]. Finally, parents were asked to indicate how often (*never, a few times, many times*) their son/daughter played in the listed social conditions (alone, with parents, with brother/sister, in an internet connection with others). 

The second section of the VGS-P is composed of nine items, each one developed in order to reveal one of the nine symptoms listed in DSM-5, provided on a 3-point Likert scale: 0 (*never*), 1 (*few times*), and 2 (*many times*). The scoring system was developed by applying item response theory (IRT) in order to have a measure of GD that takes into account the severity of each symptom described by the items [61]. Thus, the total score represents a weighted score that allows for the classification of *non-problem*, *at-risk*, and *problem video gamers*.

### 2.4. Parents’ Video Gaming Behavior

Then, parents were asked to report if they had used VGs during the isolation period [*yes*, *no*]; based on the answer, parents were classified into *video gamers* and *non-video gamers*. In the same way, parents were asked to indicate if they used the internet to play games, and, based on the answer, they were classified as *online video gamers* and *non-online video gamers*. For parents who reported playing VGs, they were asked to indicate the types of VG used [5]. Subsequently, parents had to declare how often (*never, a few times, many times*) they used the listed devices (computer, tablet, console, smartphone) to play VGs. For the purpose of analysis, the options of *“sometimes”* and *“many times”* were collapsed in the scoring procedure in order to obtain an affirmative or negative answer regarding the use of devices; in this way, a versatility score was obtained. Then, parents were asked to report at what time of day (*just woken up, at lunch, in the afternoon, at dinner, after dinner*) they played VGs. Moreover, parents were asked to indicate how many *hours a day* and how many *days a week* they played VGs; in order to determine time spent on VGs, we calculated how many *hours a week* they played VGs [62]. Finally, parents were asked to indicate how often (*never, a few times, many times*) they played in the listed social conditions (alone, with partners, with friends, with son/children). 

### 2.5. Parental Video Gaming Monitoring and Parental Knowledge of their Son/Daughter’s Life

Subsequently, they were asked to answer questions related to their knowledge of their son/daughter and the monitoring of their video gaming habits, always referring to the period of lockdown. To investigate parental knowledge, The *Parental Knowledge* subscale of the *Parental Monitoring Scale* [63,64] was used. The subscale consists of nine items that investigate parental awareness of their son/daughter’s activities, his/her friends, and the places he/she usually goes. Given the peculiarity of the lockdown period, only five items with content that could be applied to the situation were selected. Moreover, the content of some items was modified in order to make it more appropriate to the current time and associated experiences. For example, the item *“Do you know which friends your son/daughter hangs out with in his/her free time?”* was modified to *“Do you know which friends your son/daughter connects with or phones?”*; the item *“What does your son/daughter usually do after school?”* was modified to *“What does your son/daughter usually do after online lessons?”.* Cronbach’s alpha for this sample was 0.89.

Concerning parental monitoring, since, to the best of our knowledge, there is no available tool investigating parental monitoring in relation to video game behavior addressed to parents, five ad hoc items were developed by referring to the period of lockdown. Items were evaluated on a 5-point Likert scale, ranging from 1 (*totally false*) to 5 (*totally true*), and investigated parental monitoring of their child’s video gaming habits. Examples of the items are: *“I allow my son/daughter to play only at certain times of the day”* and *“I allow my son/daughter to play online”.* The scale resulted in being unidimensional (TLI = 0.939, CFI = 0.970, RMSEA = 0.052, 90% CI [0.009, 0.093]). All the factor loadings were significant at the 0.001 level and ranged from 0.36 to 0.62. Cronbach’s alpha was adequate considering the low number of items: 0.60 (95% CI [0.54, 0.64]).

### 2.6. Statistical Analyses

First, we reported a descriptive analysis of video gaming behavior. In order to comprehensively analyze video gaming, specifically during the lockdown, we considered children and adolescents separately and focused on male and female participants in each age group. We also described the parents’ video gaming habits. 

Then, in order to explain video gaming behavior in relation to parental influence, as a preliminary step, bivariate correlations between the offspring’s gender and age, parental knowledge of the son/daughter’s life, the parents’ time dedicated to gaming, parental video gaming monitoring, and time spent on VGs by the offspring and their GD symptoms were calculated. In order to control for possible relationships among the variables described above and the parent’s gender, we also took into account this variable in the correlation matrix. Subsequently, to investigate our hypothesis on the mechanisms underlying the relationships among the variables, we conducted a path analysis with AMOS 16 software (IBM SPSS Statistics, Armonk, NY, USA) [65] using maximum likelihood estimation. The presence of meditated effects among the variables was investigated through the test of indirect effects [66]. In AMOS, the bootstrap confidence interval method to define the confidence intervals for indirect effects [67] was implemented. In mediation analysis, bootstrapping is used to generate an empirically derived representation of the sampling distribution of the indirect effect, and this empirical representation is used for the construction of a confidence interval for the indirect effect. The 90% bias-corrected confidence interval percentile method was implemented using 2000 bootstrap samples. Confidence intervals for the indirect effects, which do not contain 0, are considered indicative of significant indirect effects, thus meaning the presence of a mediated effect. Several goodness-of-fit indices were used to test the adequacy of the model: the comparative fit index (CFI) [68], the Tucker–Lewis index (TLI) [69], the root mean square error of approximation (RMSEA) [70], and the standardized root mean square residual (SMRSR) [71]. CFI and TLI values equal to 0.90 or greater [68,69], RMSEA values of.08 or below [70], and SMRSR values of 0.05 or below [71] are considered indices of adequate fit.

## 3. Results

During the lockdown, 70% (*n* = 387) of the parents still worked. Among them, 69% (*n* = 245) were smart-working, i.e., they worked from home. Parents belonged to families of an average of four members (*M* = 3.69, *DS* = 0.91, range: 1–9), with about two sons/daughters (*M* = 1.83, *SD* = 0.80, range: 1–7). During the lockdown, in each house (mean m² = 114.49, *SD* = 58.52, range: 40–700) there were, on average, four cohabitants (*M* = 4.24, *SD* = 1.31, range: 2–11). There were about six open spaces in each house (e.g., terraces, gardens, vegetable gardens) (*M* = 6.47, *SD* = 3.42, range: 0–17) and four electronic devices (*M* = 4.28, *SD* = 1.06, range: 0–5). 

### 3.1. Video Gaming Behavior

Among children, the results showed that 89% (*n* = 271) were video gamers. They spent, on average, about 12 h a week on gaming (*M* = 12.15, *SD* = 9.72, range: 0.03–56). The preferred genres of VGs were first-person shooter (31%), sandbox (14%), and management (11%) games. They used, on average, about two different devices for playing VGs (*M* = 2.22, *SD* = 1.04, range: 1–5). The most used devices were smartphones (62%), followed by home game consoles and tablets (54%), handheld games consoles (29%), and computers (22%). They played VGs at about two different times on a typical day (*M* = 2.23, *SD* = 1.11, range: 1–6). The majority of children gamed during the afternoon (72%), before dinner (56%), and after lunch (43%) or in the morning (34%). Fewer children gamed after waking up (8%), before falling asleep (6%), or during dinner (2%) or lunch (1%). Concerning GD symptoms, the mean score on the VGS-P was 6.84 (*SD* = 4.89, range: 0–19.35). Based on the individual score, 46% of the children (*n* = 123) were classified as *non-problem video gamers*, 35% (*n* = 94) as *at-risk video gamers*, and 19% (*n* = 50) as *problem video gamers*. The most widespread symptoms were preoccupation (*M* = 0.97, *SD* = 0.73), lying (*M* = 0.94, *SD* = 0.80), and escape (*M* = 0.91, *SD* = 0.73). The results with regard to video gaming based on gender are reported in Table 1. 

Among adolescents, the results showed that 94% (*n* = 232) were video gamers. They spent, on average, about 18 h a week on gaming (*M* = 17.87, *SD* = 13.11, range: 0.60–70). The preferred genres of VGs were first-person shooter (35%), sports (26%), and simulation (7%) games. They used, on average, about two different devices for playing VGs (*M* = 2.47, *SD* = 0.98, range: 1–5). The most used devices to game were home game consoles (81%) and smartphones (79%), followed by computers (34%), tablets (32%), and handheld games consoles (22%). They played VGs at about two different times in a typical day (*M* = 2.56, *SD* = 1.37, range: 1–8). The majority of adolescents gamed in the afternoon (71%), before dinner (61%), or after lunch (61%). A consistent proportion of them played VGs in the morning (27%) and before falling asleep (20%). Fewer adolescents gamed after waking up (10%) or at dinner (4%) and lunch (2%). Concerning GD symptoms, the mean score at the VGS-P was 7.19 (*SD* = 5.11, range: 0–19.35). Based on these scores, 43% of the adolescents (*n* = 99) were *non-problem video gamers*, 36% (*n* = 83) *at-risk video gamers*, and 22% (*n* = 50) *problem video gamers*. The most widespread symptoms were preoccupation (*M* = 1.07, *SD* = 0.77), lying (*M* = 0.92, *SD* = 0.80), and inability to control their video gaming habits (*M* = 0.89, *SD* = 0.85). The results regarding video gaming based on gender are reported in Table 2. 

Thirty-seven percent (*n* = 204) of parents played VGs during the lockdown. On average, they spent about 7 h a week (*M* = 7.58, *SD* = 8.48, range: 0.16–69) on video gaming. 

The preferred genres of VGs were puzzles (14%), sports (13%), and board/card (9%) games. They used, on average, about one device for playing VGs (*M* = 0.90, *SD* = 1.07, range: 1–4). The most used devices to play VGs were smartphones (51%), followed by home game consoles (26%), tablets (20%), and computers (14%). The majority of parents play VGs after waking up (38%), with fewer numbers of them playing VGs at lunch (9%), during the afternoon (1%), or at dinner (1%). The majority of them are used to playing VGs alone (88%). Forty-five percent of parents gamed with their son/daughter, 20% with their partner, and 14% with their friends. 

### 3.2. Explanatory Model of Video Gaming Behavior by Parental Influences

As hypothesized, the variables taken into account were all inter-correlated (Table 3). Specifically, GD symptoms were significantly and positively related to the parents’ time dedicated to gaming and their offspring’s time spent on gaming, while significant and negative correlations were found between GD symptoms and parental video gaming monitoring and parental knowledge of their son/daughter’s life. The offspring’s time spent on VGs displayed a similar pattern of correlation with other variables. Moreover, time spent by the parents on VGs was significantly and negatively related to parental monitoring of their offspring’s video gaming habits and parental knowledge of their offspring’s life habits. Finally, a significant and positive correlation was found between parental video gaming monitoring and parental knowledge of their son/daughter’s life (Table 3). Moreover, the correlation matrix indicated that the offspring’s gender was significantly and positively correlated with the parental monitoring of their offspring’s video gaming and significantly and negatively correlated with the offspring’s time spent on VGs and GD symptoms, indicating that females were perceived as more monitored by parents than males and that males tended to spend more time on VGs and to have more GD symptoms. The offspring’s age was significantly and negatively correlated with parental knowledge of their son/daughter’s life and parental monitoring of their son/daughter’s video gaming, while a significant and positive relationship was evident between the son/daughter’s age and time spent on VGs. Thus, with increasing age, the sons/daughters tended to be less close and less monitored by parents, while they were more prone to spend time on VGs. No significant correlations with the other variables were found for the parents’ gender (Table 3). For this reason, only the offspring’s gender and age were included as covariates in the path model. 

As a subsequent step, we conducted a path analysis to test the hypothesized model. The model included parental knowledge of their son/daughter’s life and the parents’ time dedicated to gaming as two negatively correlated exogenous variables; parental monitoring of their son/daughter’s video gaming habits and time spent on VGs as the intermediary/mediator variables; and GD symptoms as the endogenous variable. In order to verify the model, controlling for socio-demographic characteristics, we then introduced gender and age in the path analysis as additional exogenous variables. Based on the correlations, we hypothesized that gender would have significant direct effects on the three endogenous variables in the model, namely, parental monitoring of their son/daughter’s video gaming habits (+), time spent on gaming (-), and the related GD symptoms (-). With regard to age, we hypothesized a significant and negative covariance with parental knowledge of their son/daughter’s life and also a significant and negative direct effect on parental monitoring over their son/daughter’s video gaming habits and a significant and positive direct effect on time spent on gaming.

**Figure 1:**
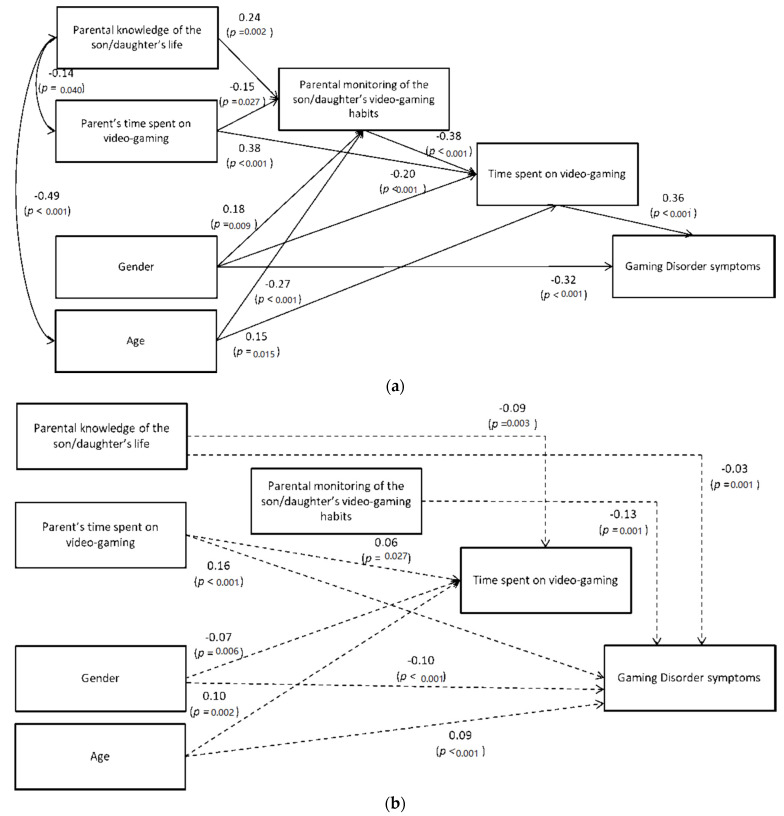
(**a**) Path model with direct effects on the variables controlling for the son/daughter’s gender and age. (**b**) Path model with indirect effects on the variables controlling for the son/daughter’s gender and age.

The model showed a good fit to the data (CFI = 0.989, TLI = 0.975, RMSEA = 0.043, SMRSR = 0.048). All coefficients were statistically significant in the expected directions. Specifically, the results revealed that parental knowledge of their son/daughter’s life and the parents’ time dedicated to gaming—negatively inter-correlated—had significant direct and positive and negative effects, respectively, on parental monitoring of their son/daughter’s video gaming habits. In turn, parental monitoring of their son/daughter’s video gaming habits was directly and negatively related to time spent on VGs, which was also affected by the parents’ time dedicated to gaming, and had a direct and positive effect on GD symptoms (Figure 1a). Moreover, the hypothesized role for gender and age was confirmed. Indeed, gender had a significant and positive direct effect on parental monitoring over their son/daughter’s video gaming habits and a significant and negative direct effect on time spent on gaming and related GD symptoms. Age had a significant and negative direct effect on parental monitoring of their son/daughter’s video gaming habits and a significant and positive direct effect on time spent on gaming. Moreover, a significant and negative covariance between parental knowledge of their son/daughter’s life and age was found (Figure 1a). 

The results also showed five significant indirect effects: from parental knowledge of their son/daughter’s life to time spent on VGs (-) and on GD symptoms (-); from the parents’ time dedicated to gaming to time spent on VGs (+) and on GD symptoms (+); and from parental monitoring on their son/daughter’s video gaming habits to GD symptoms (-) (Figure 1b). Furthermore, gender and age also exercised significant indirect effects on time spent on VGs and GD symptoms. Drilling down into the data, gender had significant and negative indirect effects on both the endogenous variables, while age had significant and positive indirect effects on them (Figure 1b).

## 4. Discussion

The aim of this study was twofold, the first being to analyze video gaming behavior in children and adolescents during the COVID-19 pandemic and the related lockdown, taking advantage of this period to better investigate the role of parents, who are most at-home due to the lockdown, with respect to their offspring’s video gaming. To this end, we tested a path model that took into account the effects of parental knowledge of their son/daughter’s life habits, parental video gaming behavior, and parental monitoring of their son/daughter’s video gaming habits and time spent on VGs on GD symptoms. 

Concerning video gaming, we found that the time spent gaming was higher than that recorded before the COVID-19 outbreak for both children [19,72] and adolescents [11,73]. As for pathological video gaming, an exceptionally high prevalence was found in adolescents and children as the rates of at-risk and problem video gamers were higher than those revealed before the global pandemic in national [74] and international [7,11,13,75] studies, in line with what has been suggested by longitudinal studies conducted before to after the spread of the COVID-19 pandemic [46,47]. It can be hypothesized that children and adolescents spent more time playing VGs during home confinement to avoid boredom and loneliness, with a consequent increase in use and, consequently, in pathological gaming. However, it is important to note that comparisons with previous rates (before the pandemic) must take into account the specificity of the COVID-19 pandemic period. Moreover, the original contribution of the present study consists of attesting to the fact that parental monitoring of the son/daughter’s video gaming habits performs a protective role in the development of GD symptoms in young people. This protective role is exercised in terms of being a mediator between specific parental antecedent variables and the children/adolescents’ gaming behavior. Indeed, the more the parent knows of the son/daughter’s life and the less time the parents themselves spend gaming, the better they are able to monitor their son/daughter’s gaming habits and, as a result, the less time their son/daughter will game; consequently, they will be less likely to develop signs of GD. Although this intermediary role of parental monitoring was valid after controlling for their offspring’s gender and age, we must note that this model has to be read in the light of some significant effects of gender and age. That is, the model suggests that female children/adolescents are more likely to be monitored by the parent with regards to their use of VGs in comparison to male children/adolescents, in line with previous studies [29], while male children/adolescents are more prone to spending a lot of time on gaming, which can have negative consequences [21,27,41,42,47]. At the same time, concerning age, the younger the son/daughter, the higher the parental knowledge [57], along with increased monitoring of the offspring’s video gaming habits [29,50,58].

Overall, the crucial role of parenting practices for juvenile video gaming is consistent with existing research showing that parental monitoring acts as a protective factor against the development of pathological gaming [42,76]. The contribution of this study is that it adds information about specific antecedents of parental monitoring and its intermediary role. In detail, this study indicates that parental monitoring is related to higher parental knowledge of the son/daughter’s life and less parental time spent gaming. Some studies have also evidenced the role of parental monitoring with respect to other addictive behaviors in adolescence, such as gambling [77], alcohol use [78], and substance use [79]. Moreover, given various parental variables consistent with this work, it has been shown that parental monitoring seems to be the most important factor that protects adolescents against problematic gambling [77]. Broadly speaking, the positive effects of parental monitoring on the physical, psychological, and social health of children and adolescents is clear [60]. Referring to VGs, as previously reported [29], parents employ various strategies, including parent–child discussion, reinforcement, rulemaking, and modeling. Parents also mediate and control their son/daughter’s interaction with VGs by restrictive mediation, namely, setting rules to control VG use in terms of the type of content and the amount of VG exposure; active mediation, specifically discussing the undesirable aspects of VG content and desirable modes of VG consumption with the son/daughter; and co-use mediation, namely, sharing the video gaming experience with children without purposeful instruction or critical discussion. The use of digital games by their sons/daughters has been found to be a major concern among parents, along with the use of restrictive strategies such as controlling content and playing time or parents and offspring playing together [80]. However, mediation through co-playing requires active participation by parents through in-depth knowledge of new technologies, which makes this parental control more difficult. Moreover, there can be different strategies of parental control over the use of VGs by their children based on the parental perception of the VGs’ effects: parents with a greater negative perception of the effects of VGs tend to apply more restrictive monitoring measures by checking ratings before giving consent to the use of particular VGs, while parents with a positive perception of VG use tend to co-play with their offspring [29]. 

## 5. Conclusions

Practical and ethical implications can be derived from this study. Parents of children and adolescents should urgently acquire and adopt effective monitoring strategies that can be helpful to avoid the development of addictive behaviors related to VGs. To this end, the first step could be to develop parental training programs designed to foster parental ability to regulate their offspring’s behavior in relation to gaming. For instance, interactive, two-way mediation seems to be the best method to have a significant effect on the frequency of VG playing [29]. It would also be useful for community initiatives to disseminate information about this issue through national health service communications. One important aim would be to educate the population about the healthy and non-healthy use of VGs. Indeed, high and repeated engagement is not necessarily associated with adverse consequences [81]. Some positive applications of VGs are increasingly attested to, such as training programs in the form of games [82] or active games, the so-called “exergames”, aimed at increasing the levels of physical activity, which offer an alternative, fun, enjoyable, and home-based mode of exercise [83]. Thus, parents should be educated about the different forms and content of VGs and the habits they stimulate. 

Especially in this pandemic period, parents must provide alternative avenues for social interaction between adolescents in order to maintain their learning motivation and to monitor and regulate their gaming time, thus minimizing addiction risks [54]. Moreover, engagement in meaningful leisure time activities may be a protective factor against video gaming involvement. For instance, playing board games, reading books for pleasure, and having other constructive interests, such as playing an instrument, drawing, and writing, could have a role in decreasing the risk of GD. These activities should be encouraged and reinforced by parents. Together with parental control strategies, those of support—such as the adaptation of parents to the needs of their children in knowing how to spot signs of discomfort and being able to engage in good listening and good communication—can represent an important factor for the protection and prevention of GD. 

Several limitations can be identified in this study. As this is a cross-sectional study, further evidence is needed to confirm the parents’ role. Moreover, although the instruments used are characterized by good psychometric properties in terms of dimensionality, internal consistency, and criterion validity, the convergent validity of the VGS-P has not been investigated, and, overall, the instruments are not standardized and are based on a hetero-evaluation. In this regard, there may be some differences between what parents reported and what the offspring really did or experienced with respect to video gaming behavior. At the same time, we did not assess the children’s and adolescents’ perception of parental monitoring, while previous research has demonstrated that parents and sons/daughters can have a different perception of parental monitoring of the use of VGs [84], with parents tending to overestimate it. Finally, this study only considers the explicative parental factors of pathological gaming. Thus, in terms of future research, it is important to confirm the present findings using a longitudinal design. It would also be interesting to replicate this study by interviewing the sons/daughters and asking them about their video gaming behavior and perceptions of parental monitoring. Moreover, these data were collected during the COVID-19 outbreak, which changed most people’s daily lives. Hence, the time spent gaming, the rates of pathological gaming, and the parents’ protective role should be assessed again to confirm the present results when life returns to normal. In conclusion, cross-national research is needed to verify this model’s generalizability to different cultural contexts where parental roles and family functioning could be different from Italy. Finally, given that multi-informant assessment is recommended for the screening and diagnosis of clinical problems at a developmental age, e.g., [85,86,87,88,89], it would be important to conduct studies in the future in which both hetero- and self-report assessment tools are integrated.

## Figures and Tables

**Table 1:** Gaming habits of children based on gender.

	Male Children		Female Children	
Prevalence of video gamers	94%		79%	
Prevalence of online video gamers	61%		39%	
Time spent on VGs (hours *per* week)	*M*	*SD*	*M*	*SD*
	13.70	9.90	7.93	7.85
Prevalence of video gamers by video game genre (only the video game genres with the highest prevalence rates are reported.)	First-person shooter games (39%)		Simulation games (15%)	
	Sports games (15%)		Sandbox games (15%)	
	Sandbox games (13%)		Fitness games (8%)	
Prevalence of video gamers by device used to play VGs	Home video game console (63%)		Smartphone (61%)	
	Smartphone (63%)		Tablet (60%)	
	Tablet (53%)		Home video game console (32%)	
	Handheld game console (34%)		Handheld game console (18%)	
	Computer (24%)		Computer (16%)	
Number of devices used to play VGs Prevalence of video gamers by time of the day	*M*	*SD*	*M*	*SD*
	2.36	1.04	1.86	1.86
	In the afternoon (74%) Before dinner (47%)		In the afternoon (71%) Before dinner (60%)	
	Before dinner (47%) In the morning (38%)		After lunch (46%) In the morning (33%)	
	After lunch (36%)		After waking up (9%)	
	After waking up (6%)		Before falling asleep (7%)	
	Before falling asleep (3%)		Before falling asleep (3%)	
	At lunch (1%)		At dinner (3%)	
	At dinner (1%)		At lunch (1%)	
Number of daily moments spent on video games	*M*	*SD*	*M*	*SD*
	2.29	1.14	2.06	1.02
Prevalence of video gamers by social partner	Alone (89%)		Alone (85%)	
	Online friends (60%)		Parents (64%)	
	Parents (53%)		Brothers/sisters (34%)	
	Brothers/sisters (51%)		Online friends (22%)	
VGS-P score	*M*	*SD*	*M*	*SD*
	7.90	4.83	3.98	3.80
Prevalence of video gamers by GD category	Non-problem gaming: 34%		Non-problem gaming: 79%	
	At-risk gaming: 44%		At-risk gaming: 13%	
	Problem gaming: 22%		Problem gaming: 8%	

Notes: VGs = video games; VGS—P = Video gaming Scale For Parents; GD = Gaming Disorder.

**Table 2:** Gaming habits of adolescents based on gender.

	Male Adolescents		Female Adolescents	
Prevalence of video gamers	100%		73%	
Prevalence of online video gamers	88%		50%	
Time spent on VGs (hours *per* week)	*M*	*SD*	*M*	*SD*
	19.11	13.16	10.99	10.62
Prevalence of video gamers by video game genre (only the video game genres with the highest prevalence rates are reported.)	Management games (41%)		Simulation games (24%)	
	Sports games (28%)		Puzzle games (18%)	
	Sandbox games (6%)		Sports games (11%)	
Prevalence of video gamers by time of the day	Home video game console (88%)		Smartphone (86%)	
	Smartphone (78%)		Home video game console (45%)	
	Computer (33%)		Tablet (38%)	
	Tablet (31%)		Computer (36%)	
	Handheld game console (22%)		Handheld game console (21%)	
Number of devices used for playing video games Prevalence of video gamers by time of the day	*M*	*SD*	*M*	*SD*
	2.52	0.96	2.26	1.06
	In the afternoon (73%) Before dinner (62%) After lunch (62%) In the morning (29%)		In the afternoon (64%) Before dinner (56%) After lunch (54%) In the morning (18%)	
	Before falling asleep (22%) After waking up (9%)		After waking up (13%) Before falling asleep (13%)	
	At dinner (5%) At lunch (2%)		At lunch (3%) At dinner (-)	
Number of daily moments spent on video games	*M*	*SD*	*M*	*SD*
	2.64	1.40	2.20	1.10
Prevalence of video gamers by social partner	Online friends (87%)		Alone (95%)	
	Alone (78%)		Brothers/sisters (46%)	
	Brothers/sisters (48%)		Online friends (41%)	
	Parents (27%)		Parents (32%)	
VGS-P score	*M*	*SD*	*M*	*SD*
	7.90	4.83	3.98	3.80
Prevalence of video gamers by GD category	Non-problem gaming: 36%		Non-problem gaming: 72%	
	At-risk gaming: 40%		At-risk gaming: 16%	
	Problem gaming: 24%		Problem gaming: 12%	

Notes: VGs = video games; VGS-P = Video Gaming Scale For Parents; GD = gaming disorder.

**Table 3:** Correlations between the offspring’s gender and age, the parents’ gender, parental knowledge of their offspring’s life, parental monitoring of their offspring’s video gaming, the parents’ time spent on VGs, and the offspring’s time spent on VGs and GD symptoms.

Variables	1.	2.	3.	4.	5.	6.	7.	8.
Gender (1 = son, 2 = daughter)	-							
2. Age	−0.07	-						
3. Parent’s gender (1 = male, 2 = female)	0.07	−0.03	-					
4. Parental knowledge of their son/daughter’s life	0.04	−0.37 ***	0.02	-				
5. Parent’s time spent on video games	−0.07	0.03	0.09	−0.19 *	-			
6. Parental monitoring of their son/daughter’s video gaming	0.14 **	−0.41 ***	0.04	0.39 ***	−0.20 **	-		
7. Time spent on VGs	−0.26 ***	0.25 ***	0.07	−0.33 ***	0.49 ***	−0.53 ***	-	
8. GD symptoms	−0.33 ***	0.05	0.03	−0.23 **	0.27 ***	−0.22 ***	0.43 ***	-
*M*	-	11.11	-	17.67	7.56	19.24	14.81	7.00
*SD*	-	3.37	-	3.44	8.48	3.47	11.76	4.99

* *p* < 0.05, ** *p* < 0.01, *** *p* < 0.001. Note: VGs = video games; GD = gaming disorder.

## Data Availability

The data presented in this study are available on request from the corresponding author. The data are not publicly available due to privacy issues.

## Appendix A

Psychometric analysis of the second section of the VGS-P confirmed the unidimensionality of the original scales (TLI = 0.949, CFI = 0.965, and RMSEA = 0.072 [95% CI = 0.057–0.089]) and good internal consistency, with Cronbach’s alpha equal to 0.89 (95% CI [0.87, 0.90]). All corrected items’ total correlations were above 0.45. As to validity, the results indicated that the VGS classification into progressive categories of gamers was discriminative with respect to video gaming involvement in terms of time spent on VGs (*F* (2, 445) = 47.59, *p* < 0.001, *η²_p_* = 0.176) and moments of the day dedicated to VGs (*F* (2, 482) = 22.64, *p* < 0.001, *η²_p_* = 0.086). Specifically, *problem video gamers* (*M* = 23.42, *SD* = 15.33) spent more time on VGs (*p* < 0.001) compared to *at-risk video gamers* (*M* = 15.68, *SD* = 9.92), who, in turn, had higher mean scores (*p* < 0.001) than *non-problem video gamers* (*M* = 10.19, *SD* = 8.59). Significant differences were also found between *problem video gamers* and *non-problem video gamers* (*p* < 0.001). At the same time, *problem video gamers* (*M* = 3.00, *SD* = 1.49) dedicated more moments of the day to VGs (*p* = 0.001) in comparison to *at-risk video gamers* (*M* = 2.49, *SD* = 1.15), who, in turn, had higher mean scores (*p* = 0.003) than *non-problem video gamers* (*M* = 2.04, *SD* = 1.05). Significant differences were also found between *problem video gamers* and *non-problem video gamers* (*p* < 0.001).

## References

[1] Kefalis C., Kontostavlou E.Z., Drigas A. (2020). The effects of video games in memory and attention. iJEP.

[2] Entertainment Software Association; 2016 Annual Report. https://www.theesa.com/wp-content/uploads/2016/12/ESAAnnualReport2016.pdf.

[3] Mentzoni R.A., Brunborg G.S., Molde H., Myrseth H., Skouverøe K.J.M., Hetland J., Pallesen S. (2011). Problematic video game use: Estimated prevalence and associations with mental and physical health. Cyberpsychol. Behav. Soc. Netw..

[4] Panagiotidi M. (2017). Problematic video game play and ADHD traits in an adult population. Cyberpsychol. Behav. Soc. Netw..

[5] Donati M.A., Chiesi F., Ammannato G., Primi C. (2015). Versatility and addiction in gaming: The number of video-game genres played is associated with pathological gaming in male adolescents. Cyberpsychol. Behav. Soc. Netw..

[6] Greenberg B.S., Sherry J., Lachlan K., Lucas K., Holmstrom A. (2010). Orientations to Video Games among Gender and Age Groups. Simul. Gaming.

[7] Gentile D. (2009). Pathological video game use among youth ages 8 to 18: A national study. Psychol Sci..

[8] Kowert R., Domahidi E., Festl R., Quandt T. (2014). Social gaming, lonely life? The impact of digital game play on adolescents’ social circles. Comput. Hum. Behav..

[9] Müller K.W., Janikian M., Dreier M., Wölfling K., Beutel M.E., Tzavara C., Richardson C., Tsitsika A. (2015). Regular gaming behavior and internet gaming disorder in European adolescents: Results from a cross-national representative survey of prevalence, predictors, and psychopathological correlates. Eur. Child. Adolesc. Psychiatry.

[10] Pontes H.M., Macur M., Griffiths M.D. (2016). Internet Gaming Disorder among Slovenian primary schoolchildren: Findings from a nationally representative sample of adolescents. J. Behav. Addict..

[11] Rehbein F., Kliem S., Baier D., Mößle T., Petry N.M. (2015). Prevalence of Internet Gaming Disorder in German adolescents: Diagnostic contribution of the nine DSM-5 criteria in a state-wide representative sample. Addiction.

[12] Donati M.A., Sanson F., Mazzarese M., Primi C. (2019). Assessing video game habits and pathological behaviour in children through a new scale: Psychometric properties of the Video-Gaming Scale—For Children (VGS-C). Psychology.

[13] Gentile D.A., Choo H., Liau A., Sim T., Li D., Fung D., Khoo A. (2011). pathological video game use among youths: A two-year longitudinal study. Pediatrics.

[14] Hamlen K.R. (2010). Children’s choices and strategies in video games. Comput. Hum. Behav..

[15] Lui D., Szeto G., Jones A. (2011). The pattern of electronic game use and related bodily discomfort in Hong Kong primary school children. Comput. Educ..

[16] Nogueira M., Faria H., Vitorino A., Silva F.G., Neto A.S. (2019). Addictive video game use: An emerging pediatric problem?. Acta Med. Port..

[17] Rideout V.J., Foehr U.G., Roberts D.F. Generation M2: Media in the Lives of 8- to 18-Year-Olds, a Kaiser Family Foundation Study 2010. Kaiser Family Foundation. https://files.eric.ed.gov/fulltext/ED527859.pdf.

[18] Hastings E.C., Karas T.L., Winsler A., Way E., Madigan A., Tyler S. (2009). Young children’s video/computer game use: Relations with school performance and behavior. Issues Ment. Health Nurs..

[19] Kovess-Masfety V., Keyes K., Hamilton A., Hanson G., Bitfoi A., Golitz D., Koc C., Kuijpers R., Lesinskiene S., Mihova Z. (2016). Is time spent playing video games associated with mental health, cognitive and social skills in young children?. Soc. Psychiatry Psychiatr. Epidemiol..

[20] (2019). European School Survey Project on Alcohol and Other Drugs Group.

[21] Gómez-Gonzalvo F., Molina P., Devís-Devís J. (2020). Which are the patterns of video game use in Spanish school adolescents? Gender as a key factor. Entertain. Comput..

[22] Homer B.D., Hayward E.O., Frye J., Plass J.L. (2012). Gender and player characteristics in video game play of preadolescents. Comput. Hum. Behav..

[23] Lobel A., Engels R.C., Stone L.L., Burk W.J., Granic I. (2017). Video gaming and children’s psychosocial wellbeing: A longitudinal study. J. Youth Adolesc..

[24] Fazeli S., Zeidi I.M., Lin C.Y., Namdar P., Griffiths M.D., Ahorsu D.K., Pakpour A.H. (2020). Depression, anxiety, and stress mediate the associations between internet gaming disorder, insomnia, and quality of life during the COVID-19 outbreak. Addict. Behav. Rep..

[25] Nielsen. 3, 2, 1 Go! Video Gaming Is at an All-Time High during COVID-19. https://www.nielsen.com/us/en/insights/article/2020/3-2-1-go-video-gaming-is-at-an-all-time-high-during-covid-19/.

[26] Flynn B. (2003). Geography of the digital hearth. Inf. Commun. Soc..

[27] King D.L., Delfabbro P.H., Billieux J., Potenza M.N. (2020). Problematic online gaming and the COVID-19 pandemic. J. Behav. Addict..

[28] Broughton M. Europe Mobile Game Revenue Hits Record High; Riot Acquires Hypixel. The Gaming Economy. https://www.thegamingeconomy.com/2020/04/17/europe-mobile-game-revenue-hits-record-high-riot-acquires-hypixel/.

[29] Shin W., Huh J. (2011). Parental mediation of teenagers’ video game playing: Antecedents and consequences. New Media Soc..

[30] Paulus F.W., Ohmann S., von Gontard A., Popow C. (2018). Internet gaming disorder in children and adolescents: A systematic review. DMCN.

[31] Jeong H., Yim H.W., Lee S.Y., Lee H.K., Potenza M.N., Lee H. (2021). Factors associated with severity, incidence or persistence of internet gaming disorder in children and adolescents: A 2-year Longitudinal Study. Addiction.

[32] Lemmens J., Valkenburg P., Peter J. (2011). Psychosocial causes and consequences of pathological gaming. Comput. Hum. Behav..

[33] Fauth-Bühler M., Mann K. (2017). Neurobiological Correlates of Internet Gaming Disorder: Similarities to Pathological Gambling. Addict. Behav..

[34] American Psychiatric Association (2013). Diagnostic and Statistical Manual of Mental Disorders DSM-5.

[35] World Health Organization (2020). International Statistical Classification of Diseases and Related Health Problems.

[36] Rumpf H.J., Achab S., Billieux J., Bowden-Jones H., Carragher N., Demetrovics Z., Higuchi S., King D.L., Mann K., Potenza M. (2018). Including Gaming Disorder in the ICD-11: The need to do so from a clinical and public health perspective. J Behav. Addict..

[37] Rehbein F., Psych G., Kleimann M., Mediasci G., Mößle T. (2010). Prevalence and risk factors of video game dependency in adolescence: Results of a German nationwide survey. Cyberpsychol. Behav. Soc. Netw..

[38] Brunborg G.S., Mentzoni R.A., Melkevik O.R., Torsheim T., Samdal O., Hetland J., Andreassen C.S., Palleson S. (2013). Gaming addiction, gaming engagement, and psychological health complaints among Norwegian adolescents. Media Psychol..

[39] Pápay O., Urbán R., Griffiths M.D., Nagygyörgy K., Farkas J., Kökönyei G., Felvinczi K., Oláh A., Elekes Z., Demetrovics Z. (2013). Psychometric properties of the Problematic Online Gaming Questionnaire Short-Form and prevalence of problematic online gaming in a national sample of adolescents. Cyberpsychol. Behav. Soc. Netw..

[40] Haagsma M.C., Marcel E.P., Oscar P. (2012). The Prevalence of Problematic Video Gamers in The Netherlands. Cyberpsychol. Behav. Soc. Netw..

[41] Bonnaire C., Phan O. (2017). Relationships between parental attitudes, family functioning and Internet gaming disorder in adolescents attending school. Psychiatry Res..

[42] Chiu S.I., Lee J.Z., Huang D.H. (2004). Video game addiction in children and teenagers in Taiwan. Cyberpsychol. Behav..

[43] Guido C.A., Amedeo I., Avenoso F., Bruni J., Zicari A.M., Loffredo L., Spalice A. (2020). Risk factors and mental health promotion strategies in children during COVID-19. Front. Public Health.

[44] Ko C.H., Yen J.Y. (2020). Impact of COVID-19 on gaming disorder: Monitoring and prevention. J. Behav. Addict..

[45] Hoenig K., Wenz S. (2020). Education, health behavior, and working conditions during the pandemic: Evidence from a German sample. Eur. Soc..

[46] Teng Z., Pontes H.M., Nie Q., Griffiths M.D., Guo C. (2021). Depression and anxiety symptoms associated with internet gaming disorder before and during the COVID-19 pandemic: A longitudinal study. J. Behav. Addict..

[47] Paschke K., Austermann M.I., Simon-Kutscher K., Thomasius R. (2021). Adolescent gaming and social media usage before and during the COVID-19 pandemic. Sucht.

[48] Zhu S., Zhuang Y., Lee P., Li J.C.M., Wong P.W. (2021). Leisure and Problem Gaming Behaviors Among Children and Adolescents During School Closures Caused by COVID-19 in Hong Kong: Quantitative Cross-sectional Survey Study. JMIR Serious Games.

[49] De Pasquale C., Chiappedi M., Sciacca F., Martinelli V., Hichy Z. (2021). Online videogames use and anxiety in children during the COVID-19 pandemic. Children.

[50] Choo H., Sim T.T., Liau A.K.F., Gentile D.A., Khoo A. (2015). Parental influences on pathological symptoms of video-gaming among children and adolescents: A prospective study. J. Child. Fam. Stud..

[51] Kwon J.H., Chung C.S., Lee J. (2011). The effects of escape from self and interpersonal relationship on the pathological use of internet games. Community Ment. Health J..

[52] Punamäki R.L., Wallenius M., Hölttö H., Nygård C.H., Rimpelä A. (2009). The associations between information and communication technology (ICT) and peer and parent relations in early adolescence. Int. J. Behav. Dev..

[53] Abelman R. (2006). Fighting the war on indecency: Mediating TV, internet, and videogame usage among achieving and underachieving gifted children. Roeper Rev..

[54] Vaala S.E., Bleakley A. (2015). Monitoring, mediating, and modeling: Parental influence on adolescent computer and internet use in the United States. J. Child Media.

[55] Ramirez E.R., Norman G.J., Rosenberg D.R.J., Kerr J., Saelens B.E., Durant N., Sallis J.F. (2011). Adolescent screen time and rules to limit screen time in the home. J. Adolesc. Health.

[56] Tang L., Darlington G., Ma D.W.L., Haines J. (2018). Mothers’ and fathers’ media parenting practices associated with young children’s screen-time: A cross-sectional study. BMC Obes..

[57] Nikken P., Jansz J. (2006). Parental mediation of children’s videogame playing: A comparison of the reports by parents and children. Learn Media Technol..

[58] Pelizzoni I., Cavallini F., Learn T., Fonticoli V., Cavallini M.C. (2019). The role of parents and the use of videogames: A Systematic Review. Media Educ. Studi Ric. Buone Prat..

[59] Gentile D.A., Reimer R.A., Amy I., Nathanson A.I., Walsh D.A., Joey C., Eisenmann J.C. (2014). Protective effects of parental monitoring of children’s media use. JAMA Pediatrics.

[60] Vondráčková P., Gabrhelík R. (2016). Prevention of internet addiction: A Systematic Review. J. Behav. Addict..

[61] Primi C., Donati M.A., Chiesi F. (2017). Video-Gaming Scale for Adolescents, VGS-A. Scala per la Misura dell’Uso dei Videogiochi negli Adolescenti [Video-Gaming Scale for Adolescents, VGS-A. A Scale to Assess Video-Gaming among Adolescents].

[62] Lemmens J.S., Patti M., Valkenburg P.M., Peter J. (2009). Development and validation of a game addiction scale for adolescents. Media Psychol..

[63] Stattin H., Kerr M. (2000). Parental monitoring: A reinterpretation. Child. Dev..

[64] Miranda M.C., Bacchini D., Affuso G. (2012). Validazione di uno strumento per la misura del parental monitoring in un campione di adolescenti italiani [Validation of a scale for measuring parental monitoring in a sample of Italian adolescents]. G. Psicol. Svilupp..

[65] Arbuckle J.L. (2007). AMOS 16.0 (Computer Software).

[66] Cheung G.W., Lau R.S. (2008). Testing mediation and suppression effects of latent variables: Bootstrapping with structural equation models. Organ Res. Methods.

[67] MacKinnon D.P., Lockwood C.M., Williams J. (2004). Confidence limits for the indirect effect: Distribution of the product and resampling methods. Multivar. Behav. Res..

[68] Bentler P.M. (1990). Comparative fit indexes in structural models. Psychol. Bull..

[69] Tucker L.R., Lewis C. (1973). A reliability coefficient for maximum likelihood factor analysis. Psychometrika.

[70] Steiger J.H., Lind J.C. Statistically Based Tests for the Number of Common Factors. Proceedings of the Annual Meeting of the Psychometric Society.

[71] Schermelleh-Engel K., Moosbrugger H., Müller H. (2013). Evaluating the fit of structural equation models: Tests of significance and descriptive goodness-of-fit measures. Psychol. Methods.

[72] Pujol J., Fenoll R., Forns J., Harrison B.J., Martínez-Vilavella G., Macià D., Alvarez-Pedrerol M., Blanco-Hinojo L., González-Ortiz S., Deus J. (2016). Video gaming in school children: How much is enough?. Annals Neuroogyl..

[73] Fumero A., Marrero R.J., Bethencourt J.M., Peñate W. (2020). Risk factors of internet gaming disorder symptoms in Spanish adolescents. Comput. Hum. Behav..

[74] Milani L., La Torre G., Fiore M., Grumi S., Gentile D.A., Ferrante M., Miccoli S., Di Blasio P. (2018). Internet gaming addiction in adolescence: Risk factors and maladjustment correlates. Int. J. Ment. Health Addict..

[75] Thomas N.J., Martin F.H. (2010). Video-arcade game, computer game and Internet activities of Australian students: Participation habits and prevalence of addiction. Aust. J. Psychol..

[76] Su B., Chengfu Y., Zhang W., Su Q., Zhu J., Jiang Y. (2018). Father–child longitudinal relationship: Parental monitoring and internet gaming disorder in Chinese adolescents. Front. Psychol..

[77] Pisarska A., Ostaszewski K. (2020). Factors associated with youth gambling: Longitudinal study among high school students. Public Health.

[78] Kaynak O., Meyers K., Caldeira K.M., Vincent K.B., Winters K.C., Arria A.M. (2013). Relationships among parental monitoring and sensation seeking on the development of substance use disorder among college students. Addict. Behav..

[79] MacPherson H.A., Wolff J., Nestor B., Frazier E., Massing-Schaffer M., Graves H., Esposito-Smythers C., Spirito A. (2021). Parental monitoring predicts depressive symptom and suicidal ideation outcomes in adolescents being treated for co-occurring substance use and psychiatric disorders. J. Affect. Disord..

[80] Kutner L.A., Olson C.K., Warner D.E., Hertzog S.M. (2008). Parents’ and sons’ perspectives on video game play. J. Adolesc. Res..

[81] Deleuze J., Long J., Liu T.Q., Maurage P., Billieux J. (2018). Passion or addiction? Correlates of healthy versus problematic use of videogames in a sample of French-speaking regular players. Addict. Behav..

[82] González C.S., Gómez N., Navarro V., Cairós M., Quirce C., Toledo P., Marrero-Gordillo N. (2016). Learning healthy lifestyles through active videogames, motor games and the gamification of educational activities. Comput. Hum. Behav..

[83] Vernadakis N., Kouli O., Tsitskari E., Gioftsidou A., Antoniou P. (2014). University students’ ability-expectancy beliefs and subjective task values for exergames. CompuT. Educ..

[84] Gentile D.A., Nathanson A.I., Rasmussen E.E., Reimer R.A., Walsh D.A. (2012). Do you see what I see? Parent and child reports of parental monitoring of media. Fam. Relat..

[85] De Los Reyes A., Augenstein T.M., Wang M., Thomas S.A., Drabick D.A.G., Burgers D.E., Rabinowitz J. (2015). The validity of the multi-informant approach to assessing child and adolescent mental health. Psychol. Bull..

[86] Gizer I.R., Waldman I.D., Abramowitz A., Barr C.L., Feng Y., Wigg K.G., Misener V.L., Rowe D.C. (2008). Relations between multi-informant assessments of ADHD symptoms, DAT1, and DRD4. J. Abnorm. Psychol..

[87] Hughes E.K., Gullone E. (2010). Reciprocal relationships between parent and adolescent internalizing symptoms. J. Fam. Psychol..

[88] Hunsley J., Mash E.J. (2007). Evidence-Based assessment. Annu. Rev. Clin. Psychol..

[89] Izzo V.A., Donati M.A., Primi C. (2019). Assessing ADHD Through the Multi-Informant Approach: The Contribution of the Conners’ 3 Scales. J. Atten. Disord..

